# Characterization of Three Novel H3F3A-mutated Giant Cell Tumor Cell Lines and Targeting of Their Wee1 Pathway

**DOI:** 10.1038/s41598-019-42611-1

**Published:** 2019-04-23

**Authors:** Christoph Lübbehüsen, Julian Lüke, Carolin Seeling, Kevin Mellert, Ralf Marienfeld, Alexandra von Baer, Markus Schultheiss, Peter Möller, Thomas F. E. Barth

**Affiliations:** 1 Institute of Pathology, Ulm University, Ulm, Germany; 2 Department of Traumatology, Ulm University, Ulm, Germany

**Keywords:** Bone cancer, Checkpoint signalling, Pharmacodynamics

## Abstract

The giant cell tumor of bone (GCTB) is a locally aggressive primary bone tumor that is composed of mononuclear stroma cells, scattered macrophages, and multinucleated osteoclast-like giant cells which cause pathologic osteolysis. The stroma cells represent the neoplastic population of the tumor and are characterized by the *H3F3A* mutation G34W. This point mutation is regarded as the driver mutation of GCTB. We have established three new stable *H3F3A* mutated GCTB cell lines: U-GCT1, U-GCT2, and U-GCT3M. MK-1775 is a Wee1-kinase inhibitor which has been used for blocking of sarcoma growth. In the cell lines we detected Wee1, Cdk1, Cyclin B1, H3K36me3, and Rrm2 as members of the Wee1 pathway. We analyzed the effect of MK-1775 and gemcitabine, alone and in combination, on the growth of the cell lines. The cell lines showed a significant reduction in cell proliferation when treated with MK-1775 or gemcitabine. The combination of both agents led to a further significant reduction in cell proliferation compared to the single agents. Immunohistochemical analysis of 13 GCTB samples revealed that Wee1 and downstream-relevant members are present in GCTB tissue samples. Overall, our work offers valuable new tools for GCTB studies and presents a description of novel biomarkers and molecular targeting strategies.

## Introduction

The GCTB is a benign but locally aggressive neoplasm. The tumor is predominantly localized in the epiphyseal region of long bones^1^. Malignant transformation is rare in GCTB occurring in less than 1%, but may rise to 6,6% after radiation therapy^2,3^. The neoplastic and mitotically active mononuclear stroma cells are characterized by a mutation in *H3F3A*^4^. In the presence of macrophage colony–stimulating factor (M-CSF) produced by macrophages the neoplastic cells recruit and activate osteolytic osteoclast giant cells by constitutive expression of receptor activator of nuclear factor kappa-Β ligand (RANKL)^1,5,6^. Surgery is used as a standard treatment^7^. However, the tumor recurs in 5 to 25% of patients^8^. Denosumab is a humanized monoclonal antibody specific for the RANKL and is used in patients with advanced disease^9^. Recently, several cases of patients with GCTB have been described in which high-grade osteosarcomas arose after treatment with denosumab^2,10,11^.

**Figure 1:**
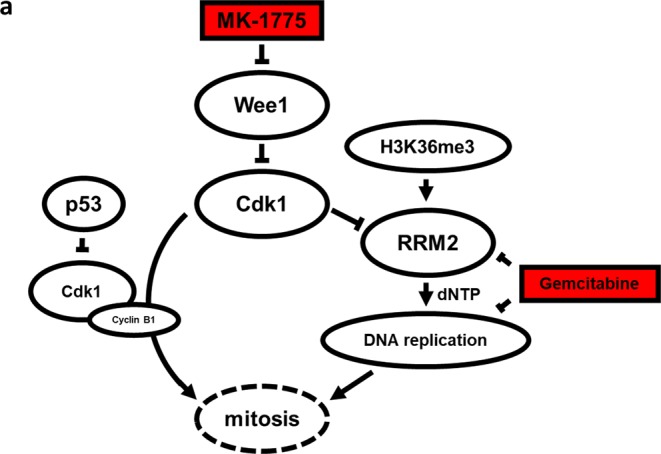
(**a**) Wee1 inactivates Cdk1 by phosphorylation at tyrosine 15^17^. Non-phosphorylated Cdk1 forms a complex with Cyclin B1 and induces mitosis^18^. Non-phosphorylated Cdk1 degrades the ribonucleotide reductase subunit Rrm2. This leads to dNTP starvation and DNA replication stress. H3K36me3 acts as an antagonist promoting Rrm2 transcription. MK-1775, as a Wee1-kinase inhibitor, leads to high Cdk1 activity and uncontrolled G2/M transition. Furthermore, MK-1775 leads to DNA replication stress and to S-phase arrest or apoptosis^19,45^. p53 protects the cell against DNA replication stress and is a potential inhibitor of Cdk1^20^. Gemcitabine inhibits DNA synthesis as a nucleoside analog of cytidine^26^.

The Wee1 pathway has been explored in several types of cancer, such as kidney cancer, colorectal cancer, melanoma, and osteosarcoma^12–16^. The Wee1 kinase controls the function of Cdk1 by phosphorylation at tyrosine 15, causing an inactivation of Cdk1 (Fig. 1a)^17^. The active state of Cdk1 forms a complex with Cyclin B1 and drives the cell into mitosis^18^. Therefore, Wee1 inhibition induces uncontrolled G_2_/M-transition and may lead to apoptosis through mitotic catastrophe, i.e., due to errors during DNA replication and repair^19^. The transition into mitosis is further controlled by p53 when cells reach the G_2_/M checkpoint with damaged DNA or when they are arrested in S-phase due to depletion of the substrates required for DNA synthesis. One mechanism by which p53 blocks cells at the G_2_/M checkpoint involves the inhibition of CDK1^20^. This suggests that a *TP53* mutation sensitizes the cell for WEE1 inhibition^13,21^. However, further studies showed that the effect is independent of the *TP53* status^16^. In an alternative pathway active Cdk1 mediates phosphorylation of Rrm2, promoting Rrm2 ubiquitylation and degradation, whereas H3K36me3 is present at the promoter of Rrm2 and recruits transcription initiation factors (TAFs). *H3F3A* mutations in GCTB are known to be associated with an increase in H3K36me3^22^. Severe Rrm2 depletion is thought to lead to dNTP starvation and to induce replication stress. For example, H3K36me3-deficient cell lines, like the kidney carcinoma cell lines A498, have been shown to be selectively killed by MK-1775 *via* dNTP starvation^12^. MK-1775, a specific Wee1 inhibitor, has been tested as a possible therapeutic option in sarcomas; e.g., Wee1 inhibition has been shown to sensitize osteosarcoma cells *in vitro* to chemotherapy or radiation at clinically feasible concentrations^15,16,23^. Compared to normal tissues, Wee1 is overexpressed in osteosarcomas^23^. In the breast cancer cell line CAL51, Wee1 is overexpressed and inhibition by MK-1775 is associated with a functional loss of Wee1 leading to cell death underlining the essential role of Wee1 in tumor cell viability^24^. In a Phase I pharmacological and pharmacodynamics study in patients with melanoma, lung cancer, ovarian cancer, breast cancer or colorectal cancer MK-1775 had a low toxicity profile both as monotherapy and in combination with DNA-damaging agents like gemcitabine (2′,2′-difluoro-2′-deoxycytidine, or dFdC)^25^. Gemcitabine is a prodrug that is di- or triphosphorylated inside the cell. The triphosphate form (dFdCTP) is a nucleoside analog of cytidine, inhibiting DNA synthesis^26^. The diphosphate form (dFdCDP) affects the enzyme ribonucleotide reductase and leads to a depletion of deoxycytidine triphosphate (dCTP) pool that potentiates the effects of the drug^26,27^.

Based on these data we have investigated the effect of the inhibitor MK-1775 and gemcitabine on the H3F3A-mutated GCTB cell lines. Here, we show that Wee1, Cdk1, H3K36me3, and Rrm2, as crucial players in cell proliferation, are detectable in both GCTB tissue samples and *in vitro*. We demonstrate that the Wee1 pathway is active in GCTB cell lines and is inhibited by a specific Wee1 inhibitor, MK-1775.

## Results

### Establishment and characterization of GCTB cell lines

**Figure 2:**
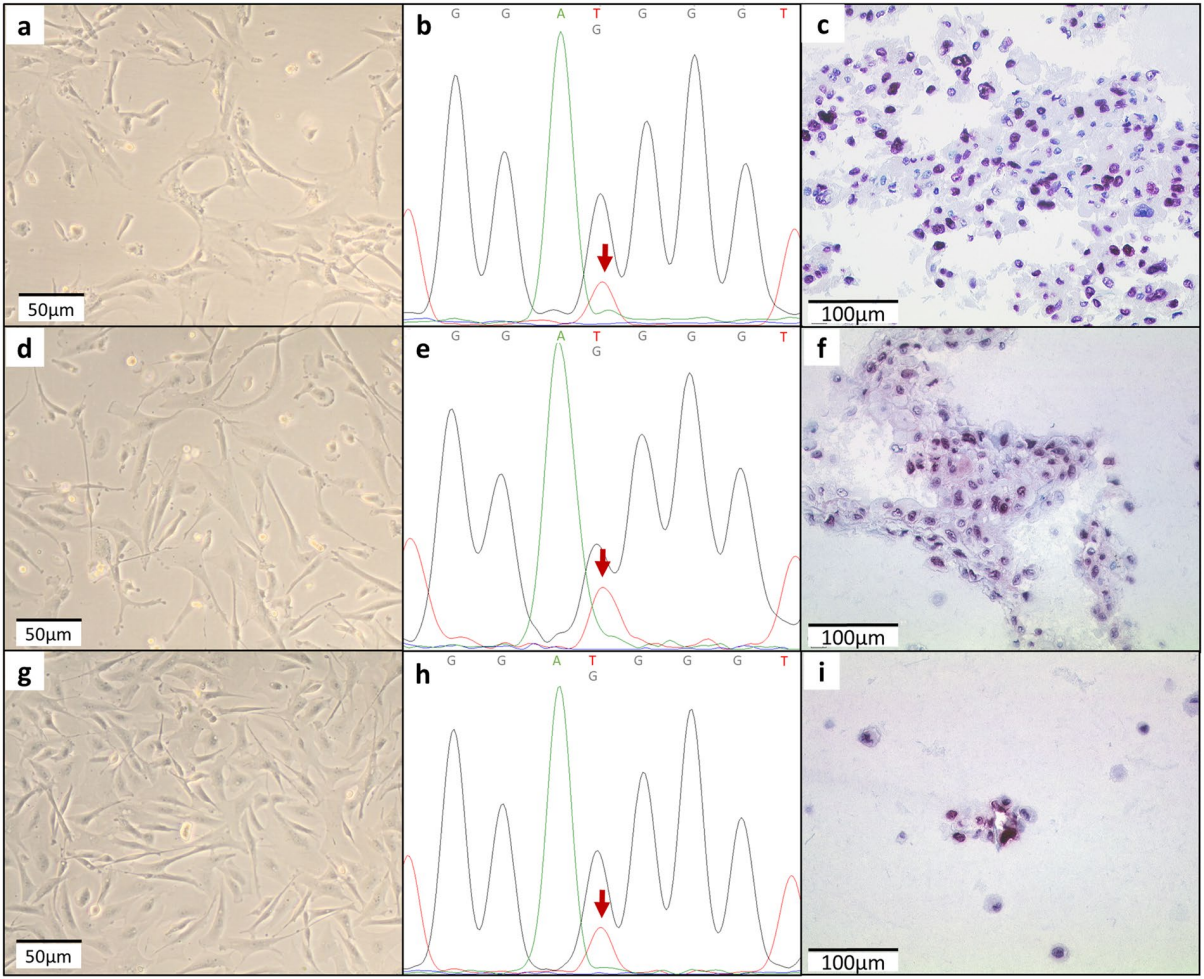
(**a**,**d**,**g**) U-GCT1, U-GCT2, and U-GCT3M morphology in cell culture. (**b**,**e**,**h**) Chromatograms of Sanger sequences of U-GCT1, U-GCT2, and U-GCT3M showing the H3F3A mutation G34W marked with the red arrow. (**c**,**f**,**i**) Anti-histone H3.3 G34W immunocytologic staining of U-GCT1, U-GCT2 and U-GCT3M.

**Figure 3:**
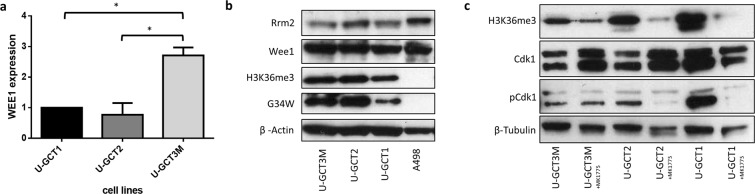
(**a**) q-PCR determination of the amount of *WEE1* expression in the U-GCT cell lines standardized to U-GCT1. (**b**) Western blot of H3.3 G34W, Wee1, H3K36me3, Rrm2 expression in the three U-GCT cell lines and A498. For the complete Western Blot see Supplementary Fig. 4. (**c**) Western blot of MK-1775-treated and untreated GCTB cell lines detecting H3K36me3, tyrosine 15 phosphorylated and non-phosphorylated Cdk1. For the complete Western Blot see Supplementary Fig. 5.

Three cell lines, U-GCT1, U-GCT2, and U-GCT3M (Table 1; Fig. 2a,d,g), were established from *H3F3A*-mutated GCTBs with typical cytologic morphology of osteoclast-like giant cells and spindle-shaped mononuclear cells (Supplementary Fig. 1a)^1^. The parental tumors harbored an *H3F3A* mutation as shown by Sanger sequencing of the relevant exon 2 and immunohistochemistry using a mutation-specific antibody G34W (Supplementary Fig. 1b)^28^. Short tandem repeats (STR) analysis of the cell lines and the parental tumor confirmed the origin of the cell lines (Supplementary Table 1). *De novo* DNA sequencing of the established cell lines revealed the *H3F3A* mutation (Fig. 2b,e,h). The *H3F3A* mutation was further confirmed by immunochemistry on formalin-fixed and paraffin-embedded cell pellets and Western blot with isolated protein from the cell lines (Figs 2c,f,i and 3b). This proved that the *H3F3A*-mutated stroma cells had been cultivated^4^, whereas the osteoclast-like giant cells did not survive further cultivation^29^. The cell lines have individual doubling times ranging from 7 to 14 days; the cell line established from the lung metastasis has the shortest doubling time of 7 days. The adherent growing cells are spindle-shaped and fibroblastic-like (Fig. 2a,d,g). These characteristics match those of the GCTB cell lines already described^30^. The *TP53* sequence analysis of the appropriate cell line DNA did not identify any relevant mutation in this tumor suppressor gene. The GCTB cell lines and all control cell lines were tested negative for mycoplasma (see Supplementary Fig. 2).

**Table 1:** Clinical data of the cohort analyzed (M = metastasis, P = primary tumor; R = recurrence).

Sample	Clinical data						IHC Expression					
	Localization	Gender	Age at diagnosis	Tissue acquisition	Status	*H3F3A* mutation	Pathway					Ki-67
							Wee1	Cdk1	Cyclin B1	H3K36me3	Rrm2	
1^U-GCT1^	proximal tibia	male	40	1999	P	positive	70%	15%	0%	60%	5%	5%
2^U-GCT2^	proximal tibia	female	17	2015	R	positive	5%	5%	<1%	80%	10%	20%
3^U-GCT3M^	lung metastasis	female	28	2016	M	positive	5%	5%	0%	90%	5%	10%
4	proximal tibia	male	49	2008	P	positive	70%	5%	0%	80%	5%	10%
5	proximal humerus	male	54	2008	P	positive	80%	35%	<1%	80%	5%	15%
6	metacarpal D1	female	41	2008	R	positive	60%	30%	0%	90%	10%	30%
7	proximal humerus	male	35	2006	P	positive	70%	10%	<1%	50%	5%	10%
8	proximal tibia	female	42	2006	R	positive	10%	5%	<1%	50%	5%	10%
9	metacarpal D4	male	49	2003	P	positive	80%	35%	<1%	80%	10%	20%
10	proximal humerus	male	24	2015	P	positive	80%	5%	<1%	50%	5%	5%
11	proximal fibula	female	50	2008	P	positive	80%	40%	<1%	90%	15%	25%
12	proximal tibia	male	50	2009	R	positive	80%	5%	0%	80%	5%	10%
13	distal femur	male	46	2014	P	positive	15%	15%	<5%	50%	15%	20%

Immunohistologic results of Wee1, Cdk1, Cyclin B1, Rrm2, H3K36me3 expression and Ki-67 index. Positive cells are given as percentages of stained cells.

### Analysis of the Wee1-pathway in GCTB

**Figure 4:**
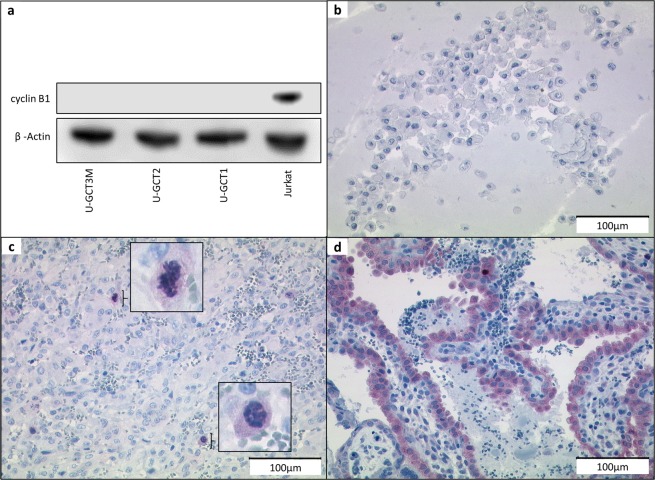
(**a**) Western blot of Cyclin B1 expression in the three U-GCT cell lines and Jurkat. For the complete Western Blot see Supplementary Fig. 6. (**b**) Cyclin B1 immunocytological staining with only a few positive cells (see Table 1; sample 11). (**c**) Cyclin B1 immunocytologic staining of U-GCT1 cell pellets. Cyclin B1. (**d**) Cyclin B1 immunohistologic staining of placental tissue as positive control.

To obtain an insight into the Wee1 signaling in GCTB, we analyzed the steps of this pathway in the U-GCT cell lines (Fig. 1a). Using quantitative polymerase chain reaction (qPCR) we quantified the expression of *WEE1* mRNA in the U-GCT cell lines. U-GCT3M showed the highest *WEE1* mRNA levels followed by U-GCT1, whereas U-GCT2 revealed the lowest amount of *WEE1* mRNA (Fig. 3a). Wee1, Cdk1, H3K36me3, and Rrm2 were detected consistently in all U-GCT cell lines, whereas A498 was negative for H3K36me3 in Western blot analyses (Fig. 3b,c). Cyclin B1 was not expressed in a detectable amount in any GCTB cell lines by Western blot or immunochemistry, whereas the Jurkat cell line was positive (Fig. 4a,b).

An *H3F3A*-mutated GCTB cohort of 13 formalin-fixed and paraffin-embedded tissues, including the parental tumors of the U-GCT cell lines, was used to confirm the findings regarding the Wee1 pathway in tissue samples and to determine the proliferation index by Ki-67 (Table 1). Wee1, Cdk1, H3K36me3, and Rrm2 were detected in the tissue samples by immunohistochemistry (Table 1; Supplementary Fig. 3). We found in GCTB tissue samples some very few intermingled Cyclin B1 positive cells with staining of the nucleus and some positive mitotic figures (Fig. 4c), as compared to the positive control (Fig. 4d). The GCTB cohort showed a Ki-67 mean value of about 14.6% and a standard deviation (SD) of 7,8%.

### Effect of MK-1775 and gemcitabine on the GCTB cell lines

**Figure 5:**
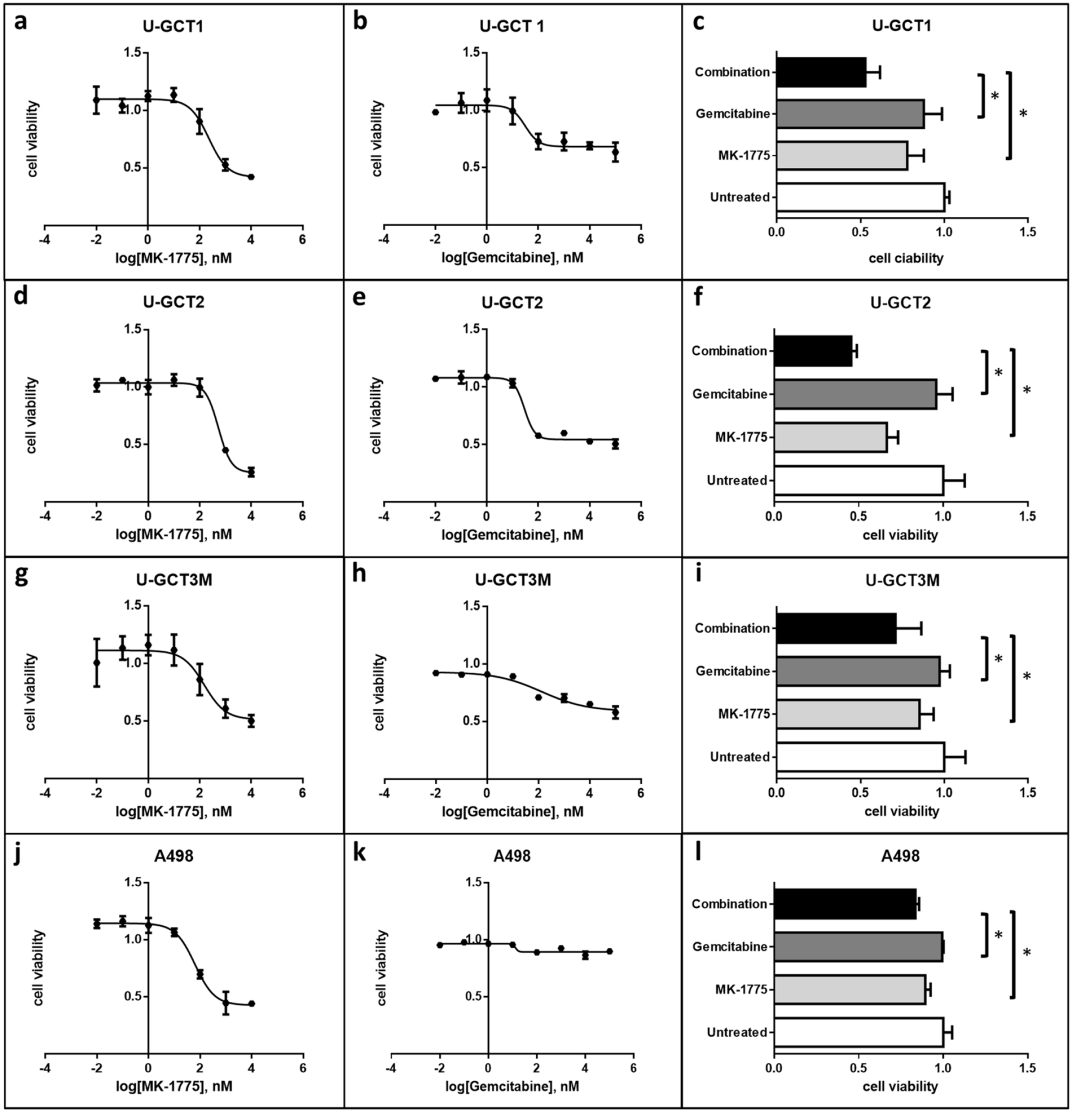
(**a**,**d**,**g**,**j**) MTS cell viability test detecting the effect of different MK-1775 concentrations on the various cell lines. (**b**,**e**,**h**,**k**) MTS cell viability test detecting the effect of different gemcitabine concentrations on the various cell lines. (**c**,**f**,**i**,**l**) MTS cell viability test detecting the effect of the IC25 single dose of MK-1775, gemcitabine and their combination on the various cell lines.

**Figure 6:**
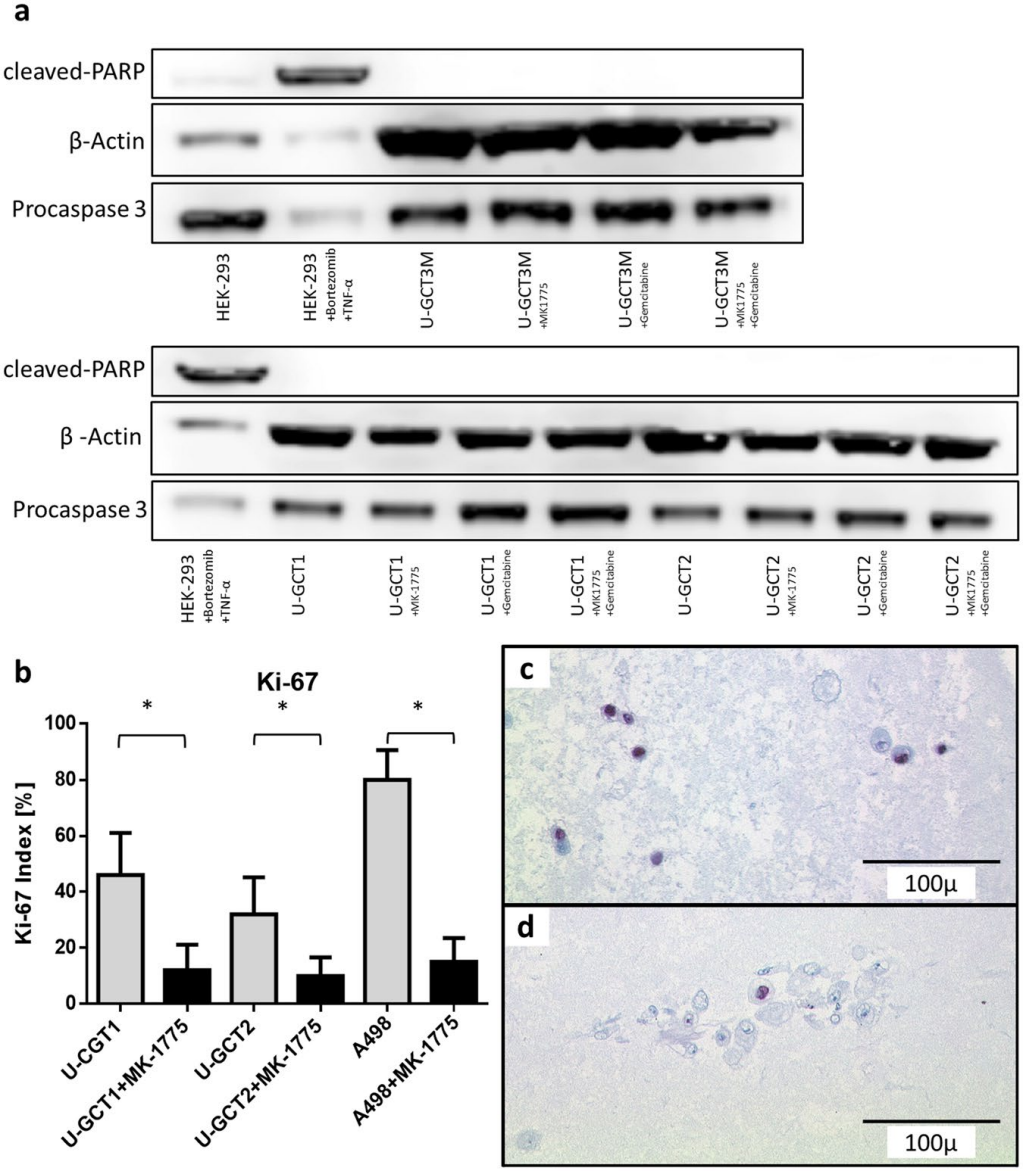
(**a**) Western blot analysis for cleaved PARP and Procaspase 3 of U-GCT cell lines as compared to HEK-293 cells (+/− stimulation with bortezomib and TNFα for 24 h). No corresponding bands are apparent in the cell lines after inhibition with 1000 nM MK-1775 or gemcitabine and the combination of both for 72 h. For the complete Western Blot see Supplementary Figs 7 and 8. (**b**) Box plots of Ki-67 index of cell pellets compared to untreated and MK-1775-treated cell lines. (**c**,**d**) Ki-67 immunocytological staining of untreated and MK-1775-treated cells of U-GCT1.

MTS assays were used to investigate the effect of Wee1 inhibition by MK-1775 on the proliferation of the GCTB cell lines. Again, we used the cell line A498 as control^12^. The IC50 value of A498 was found to be 77.89 nM (+/−23.84 nM) comparable to published data for the A498 cell line^12^. Increasing concentrations of MK-1775 led to a reduction in viable cells in all the GCTB cell lines analyzed (Fig. 5a,d,g,j). The IC50 values for these cell lines ranged from 174 to 337.8 nM (Table 2). To determine whether MK-1775 had caused a cytostatic or a cytotoxic effect on the cell lines, we used the apoptosis Western blot cocktail on isolated proteins extracted from untreated and treated cell lines (Fig. 6a). Monitoring apoptosis by analysis of cleaved PARP revealed that cleaved PARP was not detectable in the cell lines analyzed. Moreover, Ki-67 analysis showed a significant reduction in cell proliferation during MK-1775 treatment (Fig. 6b,c,d). Together, these findings argue for a cytostatic rather than a cytotoxic effect of MK-1775. We showed that Wee1 inhibition by MK-1775 leads to a decrease in the amount of H3K36me3 and a reduction in Cdk1 phosphorylation at tyrosine 15 in the GCTB cell lines, whereas Cdk1 levels remained unchanged (Fig. 3c). Treatment with an increasing concentration of gemcitabine led to decreased cell viability of all cell lines (Fig. 5b,e,h,k). The IC50 values for the U-GCTs were between 29.31 and 74.98 nM (Table 2). IC25 values for MK-1775 and gemcitabine were determined and were used either individually or as a combination of both agents (Fig. 5c,f,i,l). The combination led to a significant reduction in cell viability compared to individual treatment. However, we could not detect apoptosis even in the combination of both agents (Fig. 6a).

**Table 2:** IC50 values and standard deviations of MK-1775 and gemcitabine in different cell lines.

cell line	MK-1775 [nM]		Gemcitabine [nM]	
	IC50	SD	IC50	SD
U-GCT1	232,1	15,03	47,14	41,28
UGCT2	337,8	77,92	29,31	7,56
U-CT3M	174	85,6	74,98	60,23
A498	77,89	23,84	21,37	17,92

## Discussion

In the current study we characterized three newly generated *H3F3A* mutated GCTB cell lines including the first stable cell line of a GCTB lung metastasis. Since all these cell lines carry the *H3F3A* mutation, we concluded that these cell lines originate from neoplastic mononuclear stroma cells^4^. The Wee1 pathway has been analyzed in several tumor types. High Wee1 expression is associated with poor prognosis in pediatric high-grade gliomas, malignant melanoma, and colorectal cancer^14,31,32^. Wee1 inhibits the G_2_/M transition through inactivation of Cdk1 by phosphorylation at tyrosine 15^17^. Consistently, our data also revealed a reduction in Cdk1 phosphorylation at tyrosine 15 in the GCTB cell lines by MK-1775 mediated Wee1 inhibition^16,33^. MK-1775 was used in several preclinical studies for various cancer types, such as osteosarcoma and colorectal adenocarcinoma. *TP53* mutation has been described as a sensitizing factor for inhibition with MK-1775^13^. In line with published data in GCTBs, we found no point mutations in *TP53*^34^. Therefore, our results confirm data from osteosarcoma cell lines showing that the efficacy of MK-1775 inhibition is independent of the *TP53* mutation status^16^. Moreover, we show that the GCTBs express Wee1 and other relevant players of this pathway *in vitro* and in tissue samples. Higher Wee1 expression is associated with higher sensibility for MK-1775^24^. We can confirm this, since UGCT3M shows a significantly higher expression of *WEE1* and the lowest IC50 value of the GCTB cell lines. Thus, the inhibitory effect of MK-1775 is linked to the amount of Wee1. A further factor that may influence the Wee1 inhibition is the presence of H3K36me3. *H3F3A* mutations in GCTB are linked to an increase of H3K36me3^22^, although the functional role of H3K36me3 in GCTBs is unknown. The higher IC50 values for MK-1775-mediated growth inhibition for GCTB cell lines as compared to the H3K36me3-negative cell line A498 is interesting. This observation together with the decrease in H3K36me3 levels upon Wee1 inhibition supports the notion that H3K36me3 has a functional role in this pathway and may have a cell-protecting effect^12^. Compared with other bone tumors, GCTBs have a lower proliferation rate as detected by a low Ki-67 index^35,36^. Cyclin B1 acts in a dose-dependent manner. During the S-phase, cyclin B1 increases and forms a complex with Cdk1, leading to mitosis^37^. These data and the fact that Cyclin B1 in our results across from others is expressed in very low amounts in the GCTBs^36^ rise the hypothesis that, in our cell system, Wee1 inhibition eventually may cause dNTP starvation rather than an uncontrolled G_2_/M-transition^12^. Gemcitabine has been proven to induce growth inhibition in osteosarcomas that were refractory to standard chemotherapy^38^. Moreover, Wee1 inhibition has been shown to enhance the effect of DNA-damaging agents, such as gemcitabine, in osteosarcomas^13,15^. We show that the combination of both agents reveals a further significant reduction in cell proliferation compared to the IC25 single application, including in GCTB cells. This combinatorial effect may be explained by the reduced levels of dNTP causing Wee1 inhibition^12^ and the diminished ribonucleotide reductase activity triggered by dFdCDP, leading to a depletion of the dCTP pool^26,27^. Equally dFdCTP as a nucleoside analog of cytidine inhibits DNA synthesis^26^.

First-line treatment for GCTB is surgical curettage of the lesion^7^. In the event of recurrence or at advanced stages, denosumab as anti-RANK ligand agent has been introduced as a therapeutic concept for GCTB^9^. The effect of denosumab seems to be limited, since it blocks the osteoclasts and not the neoplastic stroma cells^39^. In histologic analysis, denosumab-treated GCTBs showed marked giant cell depletion and new bone deposition, leading to substantial histologic overlap with other primary tumors of bone^40^. Recently, several cases of denosumab-treated GCTBs were described that showed a transformation into a high-grade osteosarcoma with poor prognosis^2,10,11^. These facts underline the need for novel treatment strategies. In a Phase I study on patients with advanced solid tumors, MK-1775 was well tolerated as monotherapy and in combination with chemotherapeutic substances like gemcitabine^25^. Since we have detected Wee1, Cdk1, and H3K36me3 in the GCTB tissue samples, these proteins may serve as biomarkers for a definition of patients with GCTB eligible for a potential Wee1 inhibition-based therapy. Taken together, our data argue for an extension of Wee1 therapy to progressed GCTB patients. Therefore, the initiation of a prospective study including GCTB patients expressing the defined biomarkers of the Wee1 pathway by immunohistologic profiling, should now be considered.

## Material and Methods

### Cell culture and establishment of novel GCTB cell lines

Three novel stable GCTB cell lines were established. U-GCT1 was established from a GCTB measuring 6.0 × 6.0 × 2.0 cm, obtained from the right proximal tibia of a 40-year-old male patient in 05/1999. U-GCT2 was established from a GCTB measuring 4.5 × 4.2 × 2.0 cm obtained from the left proximal tibia of a 17-year-old female patient in 07/2015. The third cell line, U-GCT3M, was established in 03/2016 and obtained from a lung metastasis of a 28-year-old female patient with a GCTB in the left distal femur. The lung manifestation measured 3.0 × 1.3 × 1.0 cm and was localized in segment 6 of the left inferior lobe of the lung. All three patients gave their informed and written consent to the scientific use of their tumor cells. In case of the 17-year-old patient the parental authority additionally gave the informed consent. The research was approved by the local ethics committee of the University of Ulm (reference 369/17 and 372/17).

After extraction, the tumor tissue was prepared for cell culture. The tissue was minced into small pieces and partially digested with collagenase (Sigma-Aldrich, St. Louis, USA, catalog number (cat. #): C9891) 1 mg/ml diluted in 50 nM Trizma Base (Sigma-Aldrich, cat. #: RDD008) and 0.32 nM calcium chloride dihydrate (Sigma-Aldrich, cat. #: C3881), pH 7.4. Isolated cells were cultured in Nunc EasYFlask 25 cm^2^ cell culture flask (Thermo Scientific, Waltham, USA, cat. #: 156367) with Iscove’s Modified Dulbecco’s Medium with 25 mM HEPES (4:1; Lonza, Basel, Switzerland, cat. #: BE12-726F) with 10% fetal bovine serum (Biochrom AG, Berlin, Germany, cat. #: S0115), 2 mM L-glutamine (Lonza, cat. #: BE17-605E), and penicillin-streptomycin (Lonza, cat. #: DE17-602E). Cells were detached with trypsin (Lonza, cat. #: BE17-161E) before reaching a state of complete confluence. As control cells we used the commercially available cell lines A498, Jurkat, and HEK-293 (Leibniz Institute DSMZ German Collection of Microorganisms and Cell Cultures, Braunschweig, Germany). The cell line experiments were performed within 4 months after STR analysis and negative testing for mycoplasma by PCR. All cell lines were cultured at 37 °C in a 5% CO_2_ incubator applying the same protocol as mentioned above.

### Short tandem repeats (STR) analysis

DNA from the cell lines was extracted using the QIAamp DNA Mini Kit (Qiagen, Hilden, Germany, cat. #: 51304). STR analysis was performed comparing the parental tumors with the corresponding cell lines and genotyping them and the control cell lines by using a standard protocol^41^.

### Polymerase chain reaction (PCR)

Cell lines were tested for the presence of mycoplasma using PCR. PCR amplification was done using specific primers for mycoplasma DNA (primer sequences in Supplementary Table 2). The PCR reaction was carried out in a total volume of 25 µl, including 1 µl DNA or sterile water as a negative control. As positive control isolated DNA from a mycoplasma infected cell line was used. 12,5 µl OneTaq Quick-Load (New England BioLabs, Ipswich, USA, cat. #: #0486S), 1 µl of each primer (5 pmol/µl) and 9, 5 µl of sterile water. PCR was performed using the LabCycler Compact (SensoQuest, Göttingen, Germany, cat. #: 95-3000-048) with a cycling protocol as follows: 10 minutes initial denaturation at 95 °C followed by 55 cycles with 30 seconds at 95 °C, 30 seconds at 50 °C, 30 seconds at 68 °C and final extension at 72 °C for 10 minutes. The PCR products were resolved on a 2% agarose gel.

### Gene sequencing

Gene sequencing (Sanger) was performed according to our diagnostic standard protocol^28^. The tumor tissues and the corresponding cell lines were analyzed for the mutational status of *H3F3A*. The cell line A498 were used as negative controls. The graphs were generated with FinchTV 1.4.0 (Geospiza Inc., Seattle Washington, USA).

### *TP53* sequencing analysis

Exons of *TP53* were amplified using a Multiplex PCR Kit (Qiagen, cat. #: 206143). Subsequently, PCR products were sequenced using the MiSeq-Sequencer (Illumina, San Diego, USA, cat. #: Y-410-1003) with a coverage of 5000x. The resulting sequences were compared to an hg19 as the reference genome using the MiSeq Reporter Software. The non-synonymous aberration was checked using the Integrated Genome Viewer. Genomic areas were reviewed for clinically relevant *TP53*-point mutations using http://cancer.sanger.ac.uk/cosmic.

### Real-time polymerase chain reaction (qPCR)

RNeasy Mini-Kit (Qiagen, cat. #: 74104) was used for extracting total RNA. A NanoDrop 2000 spectrophotometer (Thermo Scientific, cat. #: ND-2000) was used to control the RNA quality and quantity. Omniscript Reverse Transcription Kit (Qiagen, cat. #: 20511) was used to synthesize single-strand cDNA from total RNA. QuantiTect SYBR Green PCR Kit (Qiagen, cat. #: 204143) and the Light-cycler Rotor Gene Q (Qiagen, cat. #: 9001560) were used to perform the qPCR. The expression of the WEE1 gene was analyzed using the appropriate QuantiTect Primer Assays (Qiagen, cat. #: QT00038199). β-actin and GAPDH served as housekeeping genes (primer sequences in Supplementary Table 2). The PCR reaction was performed as follows: the initial polymerase activation step was 95 °C for 15 minutes, followed by 60 cycles of 94 °C for 15 seconds, at 55 °C (*WEE1*) or 58 °C (*β-Actin* and *GAPDH*) for 30 seconds and at 72 °C for 30 seconds. All experiments were performed in triplicate.

### Protein isolation and Western blotting

Protein isolation and Western blots were performed using standard protocols as described elsewhere^41^. The following primary antibodies were used: R2 (Santa Cruz Biotechnology, Dallas, USA, cat. #: sc-10844, 1:1000), Wee1 (Santa Cruz Biotechnology, cat. #: sc-9037, 1:1000), Anti-Histone H3.3 G34W (RevMAb Biosciences, South San Francisco, USA, cat. #: 31-1145-00, 1:1000), Histone H3K36me3 antibody (Active Motif, Cambridge, UK, cat. #: 61021, 1:1000), Cdc2 p34 (Santa Cruz Biotechnology, cat. #: sc-54, 1:500), Phospho-cdc2 Tyrosine 15 (Cell Signaling, Denver, USA, cat. #: #9111, 1:500), and Cyclin B1 (Santa Cruz Biotechnology, cat. #: sc-245, 1:200). As secondary antibody we used: Anti-Rabbit IgG (Sigma-Aldrich, cat. #: A-9169,1:10000), rabbit anti-goat IgG-HRP (Santa Cruz Biotechnology, cat. #: sc-2768, 1:10000) and goat anti-mouse IgG-HRP (Santa Cruz, cat. #: sc-2005, 1:5000). Anti-β-Actin antibody (Sigma-Aldrich, cat. #: A2228, 1:100000) or Anti-β-Tubulin (Sigma-Aldrich, cat. #: T4026,1:2000) were used to detect β-actin or β-tubulin as a housekeeping protein.

### MTS proliferation assay

For inhibition assays, MK-1775 (MedChem Express, Monmouth Junction, USA, cat. #: HY-10993) was diluted in DMSO (Sigma Aldrich, cat. #: D4540) and gemcitabine hydrochloride (Sigma-Aldrich, cat. #: G6423) was diluted in PBS (Lonza, cat. #: 17-516).

For MTS cell proliferation assays, 15000 cells/cm^2^ of the cell lines were seeded in Nunclon Delta 96-Well MicroWell Plates (Thermo Scientific, cat. #: 163320). An initial incubation at 37 °C and 5% CO_2_ for 24 hours ensured complete adherence and spreading of the cells. The cells were then incubated for 72 hours under the above-mentioned cell culture conditions at eight different concentrations of the agents increasing in decimal power from 0.01 nM to 100 µM. Untreated cells served as controls. Five wells were prepared for each treatment. As the next step, 10 µl CellTiter 96 AQueous One Solution Cell Proliferation Assay (MTS) (Promega, Madison, USA, cat. #: G3580) were added to all wells and the absorption was measured after 3 hours using the Biokinetics Microplate Reader (Bio-Tek Instruments, Winooski, USA, cat. #: EL340) with a 490 nm filter. The results were calibrated in Excel 2016 (Microsoft, Redmond, USA, cat. #: SWR0616). The average of the medium data was subtracted from the other results and the inhibition data were then divided by the average of the dilution solution only-treated cells. The combination effect of MK-1775 and gemcitabine were established by comparing the detected IC25 values of the agents in single-dose and combination as described above.

For apoptosis, Western blot and Ki-67 analysis, the cell lines were cultivated in 2 ml medium in a Nunc EasYFlask 25 cm^2^ Cell Culture Flask (Thermo Scientific, cat. #: 156367) for 24 hours. The cells were then incubated with 3 mM of the MK-1775, gemcitabine, or both agents in 1 ml medium for 72 h at a concentration of 1 mM. HEK293 cells were treated with 5 ng/ml bortezomib (Santa Cruz, cat. #: sc-217785), diluted in DMSO and 50 ng/ml TNF-α (PeproTech US, Rocky Hill, USA, cat. #: 300-01 A) diluted in medium for 24 hours and were used as positive controls^42^. Protein isolation and Western blot were conducted using the above-mentioned protocol and subsequently using the primary and secondary antibodies of the apoptosis Western blot cocktail (Abcam, Cambridge, UK, cat. #: ab136812) as previously described. Formalin-fixed and paraffin-embedded cell pellets were prepared using standard protocols. Ki-67 (Dako, Glostrup, Denmark, cat. #: M7240, 1:200) stainings were performed as described below and evaluated on 3 paraffin sections of cell pellet preparations. At least 100 cells were counted on each cytoblock section. Untreated controls were included in every experiment. All experiments were repeated at least three times.

### Giant cell tumor tissue bank

GCTB tissue samples with *H3F3A* mutation from 13 patients were available from the Ulm tissue bank (median age: 40.39 years; range: 17–54 years; 8 males, 5 females). The parental tumors of the cell lines were included (Table 1). Eight patients had localized disease, 4 had recurrences and one patient had metastatic disease. The diagnosis was based on histologic subtyping according to the WHO^1^. The samples were pseudonymized according to the German law for correct usage of archival tissue for clinical research^43^. Approval for this procedure was obtained from the local Ethics Committee (vote for usage of archived human material 03/2014) and was in compliance with the ethical principles of the WMA Declaration of Helsinki^44^.

### Immunochemistry

The alkaline phosphatase/RED detection system (Dako, cat. #: K5005) was used for immunohistochemistry and immunochemistry on formalin-fixed and paraffin-embedded tissue or cell pellets via the avidin-biotin-complex-method. The following antibodies were used: Anti-Histone H3.3 G34W (RevMAb Biosciences, cat. #: 31-1145-00, 1:400), Wee1 (Santa Cruz Biotechnology, cat. #: sc-037, 1:25), Cdc2 p34 (Santa Cruz Biotechnology, cat. #: sc-54, 1:200), Cyclin B1 (Santa Cruz Biotechnology, cat. #: sc-245, 1:25), Histone H3K36me3 Antibody (Active Motif, cat. #: 61021, 1:8000), R2 (Santa Cruz Biotechnology, cat. #: sc-10844, 1:100), and Ki-67 Antigen (Dako, cat. #: M7240, 1:200). As retrieval methodes all sides were pretreated by a steamer in EDTA pH 8. Consensus evaluation was done on multihead microscope (CL, TFB, PM).

### Statistics

For statistical analysis, two-sided t-tests were performed. A p-value ≤ 0.05% was considered as significant. The graphs were generated with Prism Version 6.01 (Graphpad Software, La Jolla, USA, cat. #: 1820).

All methods were performed in accordance with the relevant guidelines and regulations of good scientific practice of the University of Ulm and the Deutsche Forschungsgemeinschaft (DFG): 10.1002/9783527679188.oth1.

## Supplementary information

Supplementary Data
