# Supplementary material for: Characterization of Three Novel H3F3A-mutated Giant Cell Tumor Cell Lines and Targeting of Their Wee1 Pathway
Source: Sci Rep. 2019 Apr 23;9:6458. doi: 10.1038/s41598-019-42611-1 (PMC6478864; doi:10.1038/s41598-019-42611-1)
Supplement: Supplementary file 1 — Supplementary Data [file 41598_2019_42611_MOESM1_ESM.pdf]

Characterization of Three Novel H3F3A-mutated Giant Cell Tumor Cell Lines and Targeting of Their Wee1 Pathway

Christoph Lübbehüsen[1], Julian Lüke[1], Carolin Seeling[1], Kevin Mellert[1], Ralf Marienfeld[1], Alexandra von Baer[2], Markus Schultheiss[2], Peter Möller[1*], and Thomas FE Barth[1]

[1]Institute of Pathology, Ulm University

[2]Department of Traumatology, Ulm University

Supplementary Data

Supplementary Table 1

	U-GCT1		U-GCT2		U-GCT3M	
	Parental Tumour	Cell Line	Parental Tumour	Cell Line	Parental Tumour	Cell Line
STR-Marker	Allele	Allele	Allele	Allele	Allele	Allele
AMEL	X;Y	X;Y	X;X	X;X	X;X	X;X
D3S1358	15;17	15;17	15;18	15;18	12;16	12;16
D8S1179	10;11	10;11	10;16	10;16	13;13	13;13
TPOX	8;11	8;11	11;11	11;11	8;11	8;11
CSF1PO	10;13	10;13	10;10	10;10	10;12	10;12
Penta D		13;13	12;13	12;13	7;11	7;11
D13S317	11;13	11;13	11;13	11;13	11;12	11;12
D7S820	9;13	9;13	8;11	8;11	10;13	10;13
D16S539	11;12	11;12	11;12	11;12	9;10	9;10
Penta E		7;10	7;12	7;12	12;12	12;12
TH01	6;6	6;6	8;9.3	8;9.3	7;8	7;8
D18S51	13;13	13;13	12;13	12;13	13;16	13;16

Legend Supplementary Table 1

Short tandem repeats (STR) analysis proved that the parental tumors and corresponding cell line match.

Supplementary Table 2

**b-Actin**	
for	5’TGTGGCATCCACGAAACTAC3’
rev	5’GGAGCAATGTTGATCTTCA3’
**GAPDH**	
for	5’GCCAAAAGGGTCATCATCTC3’
rev	5’ TGTGGTCATGAGTCCTTCCA3’
**mycoplasma**	
for	5'CRCCTGRGTAGTAHRHWCAG'3
rev	5'GCGGTGTGTACAARMCCCGA'3

Legend Supplementary Table 2

Primer sequences of β-actin, GAPDH, and mycoplasma manufactured by Biomers.

Supplementary Figure 1

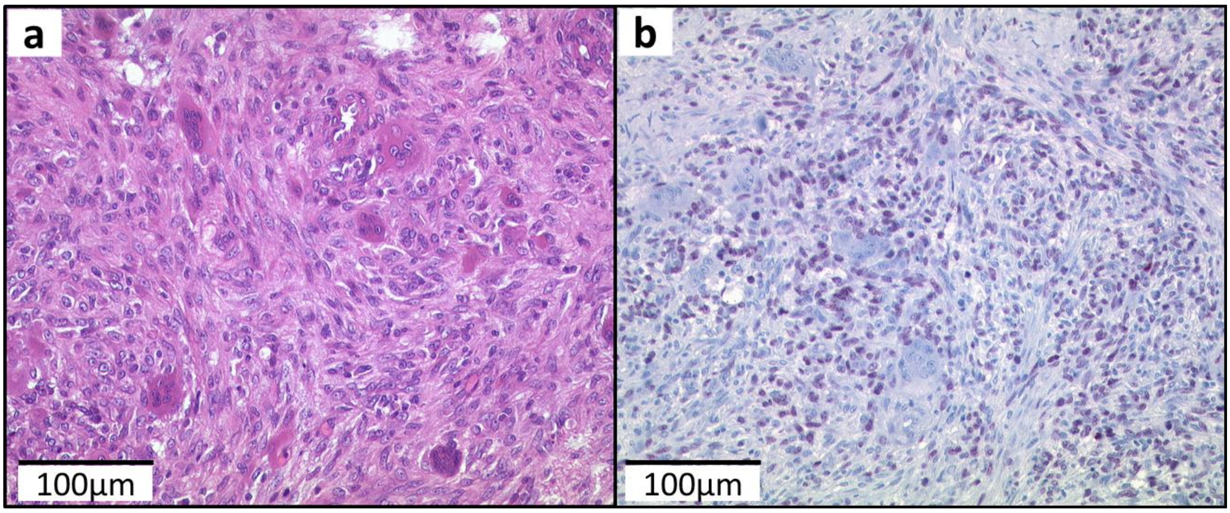

Legend Supplementary Figure 1

a: Hematoxylin and eosin staining of the parental tumor of U-GCT1.

b: Anti-histone H3.3 G34W immunohistochemical staining of the parental tumor of U-GCT1.

Supplementary Figure 2

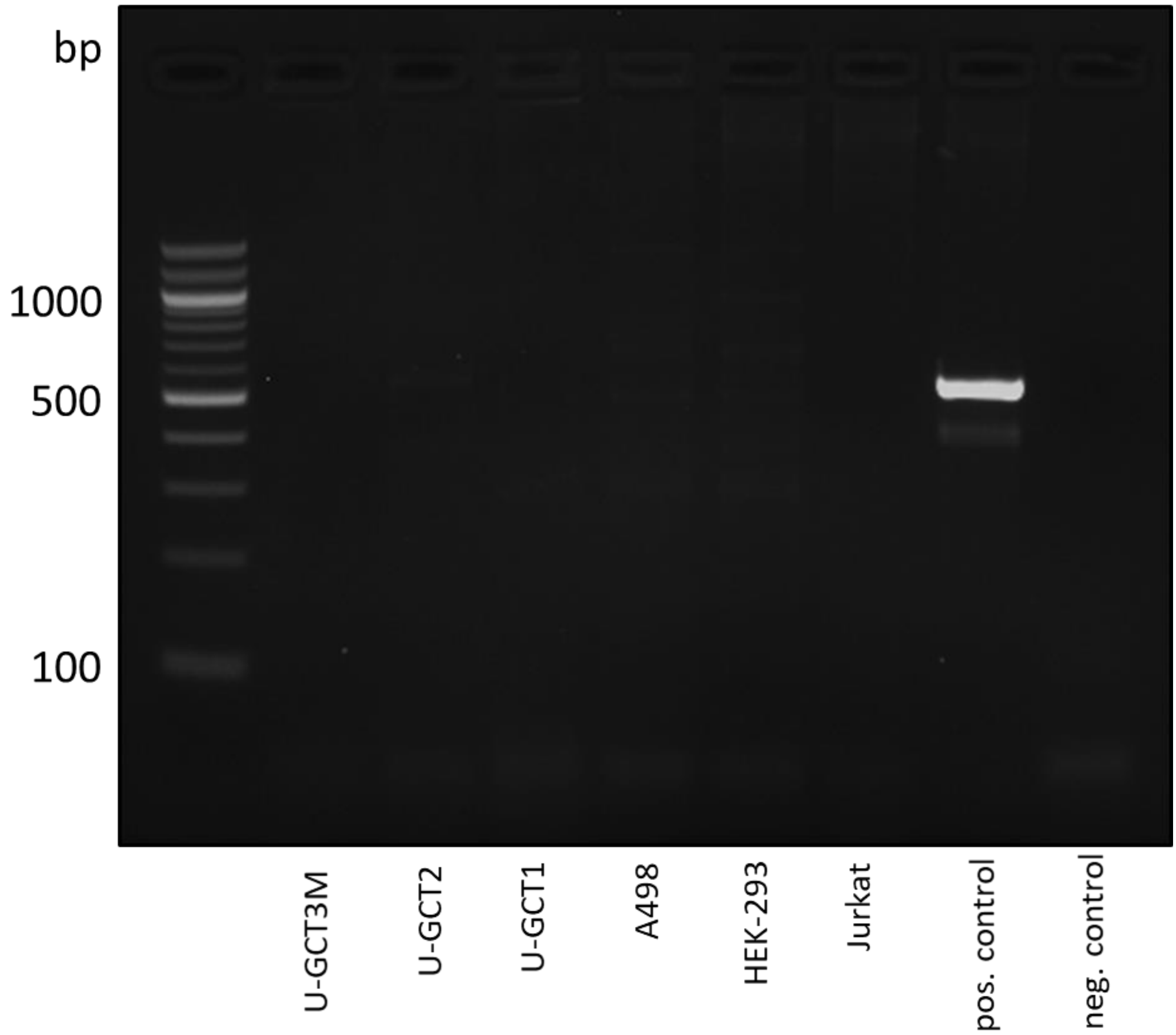

Legend Supplementary Figure 2

U-GCT3M, U-GCT2, U-GCT1, A498, HEK-293, and Jurkat cells tested negative for mycoplasma using PCR.

Supplementary Figure 3

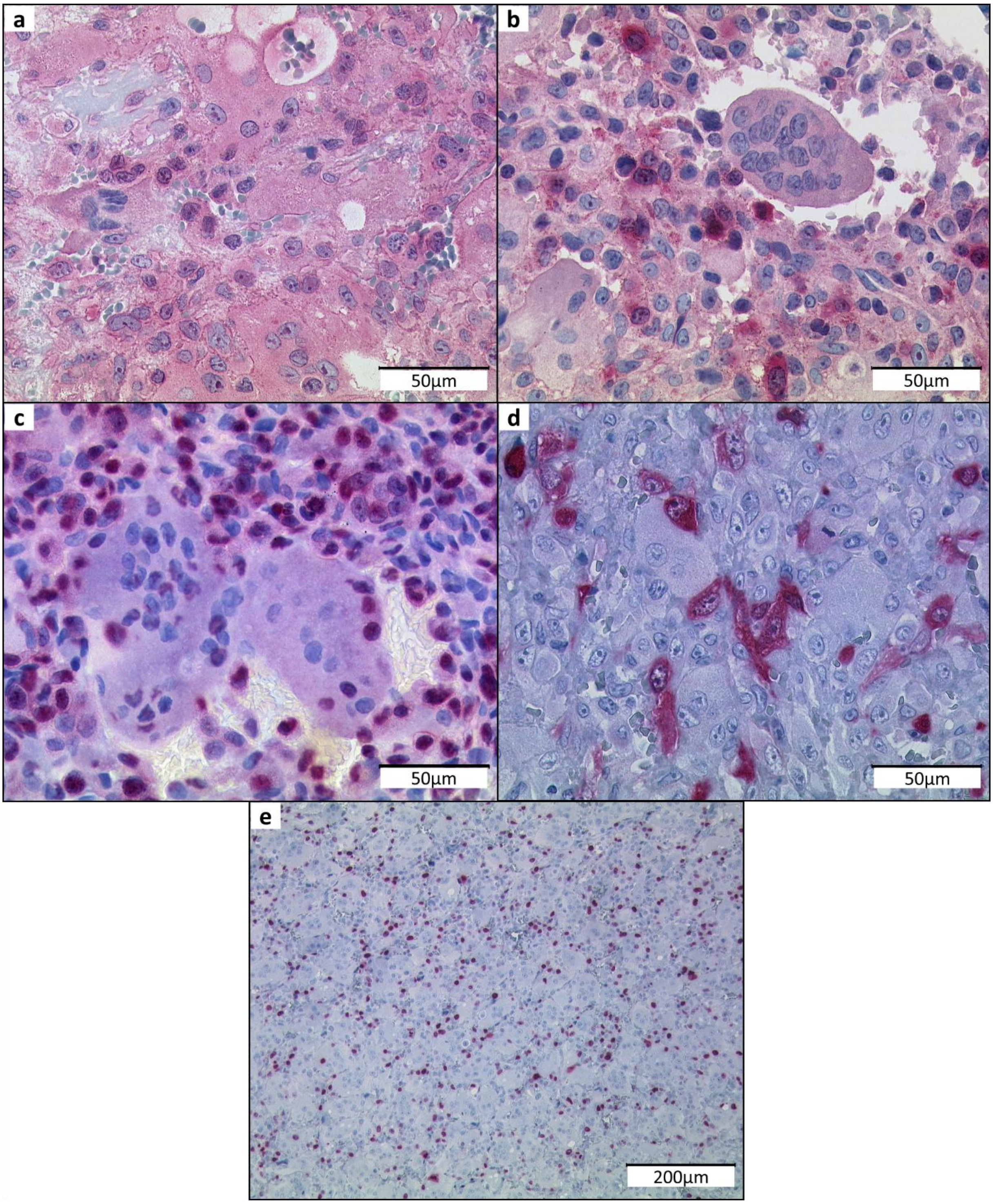

Legend Supplementary Figure 3

a, b, c, d, e: Immunohistologic staining of Wee1, Cdk1, H3K36me3, Rrm2, and Ki-67 (sample 11).

Supplementary Figure 4

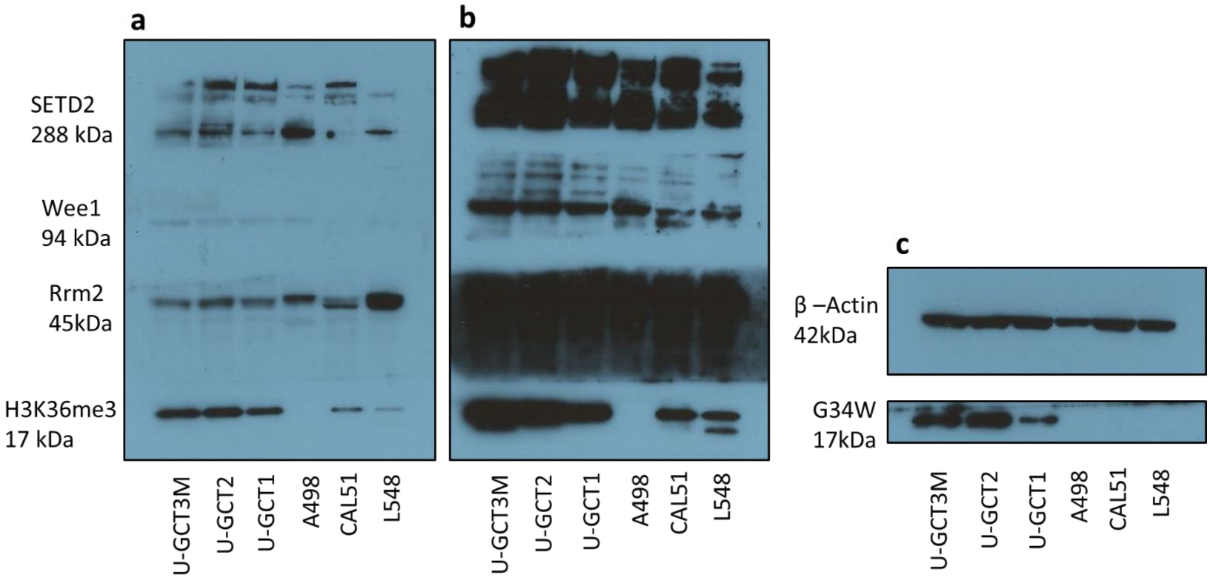

Legend Supplementary Figure 4

a, b: Whole Western-Blot detecting SETD2, Wee1, Rrm2 and H3K36me3 at two different exposure times in the U-GCT cell lines, A498, CAL51 and L548.

c: Western-Blot detecting β-Actin and H3.3 G34W, after stripping of the membrane, which were used for Supplementary Figure 7 a and b.

Supplementary Figure 5

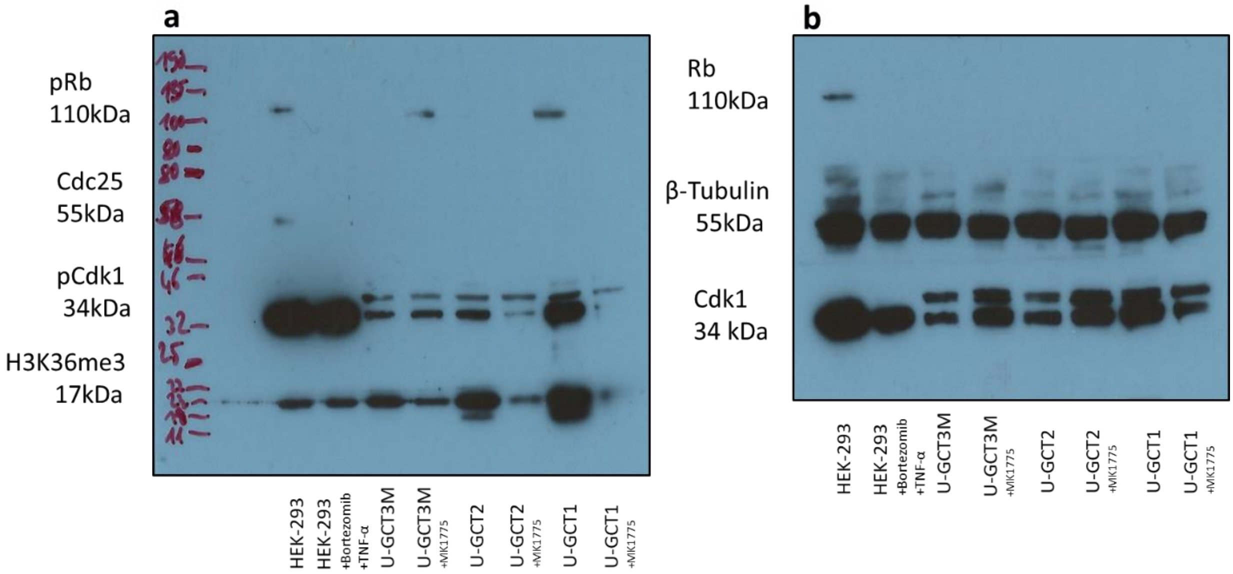

Legend Supplementary Figure 5

a: Whole Western-Blot detecting pRb, Cdc25, pCdk1 and H3K36me3 in the U-GCT cell lines and the control cell line.

b: Western-Blot detecting Rb, β-Tubulin and Cdk1, after stripping of the membrane, which were used for Supplementary Figure 8 a.

Supplementary Figure 6

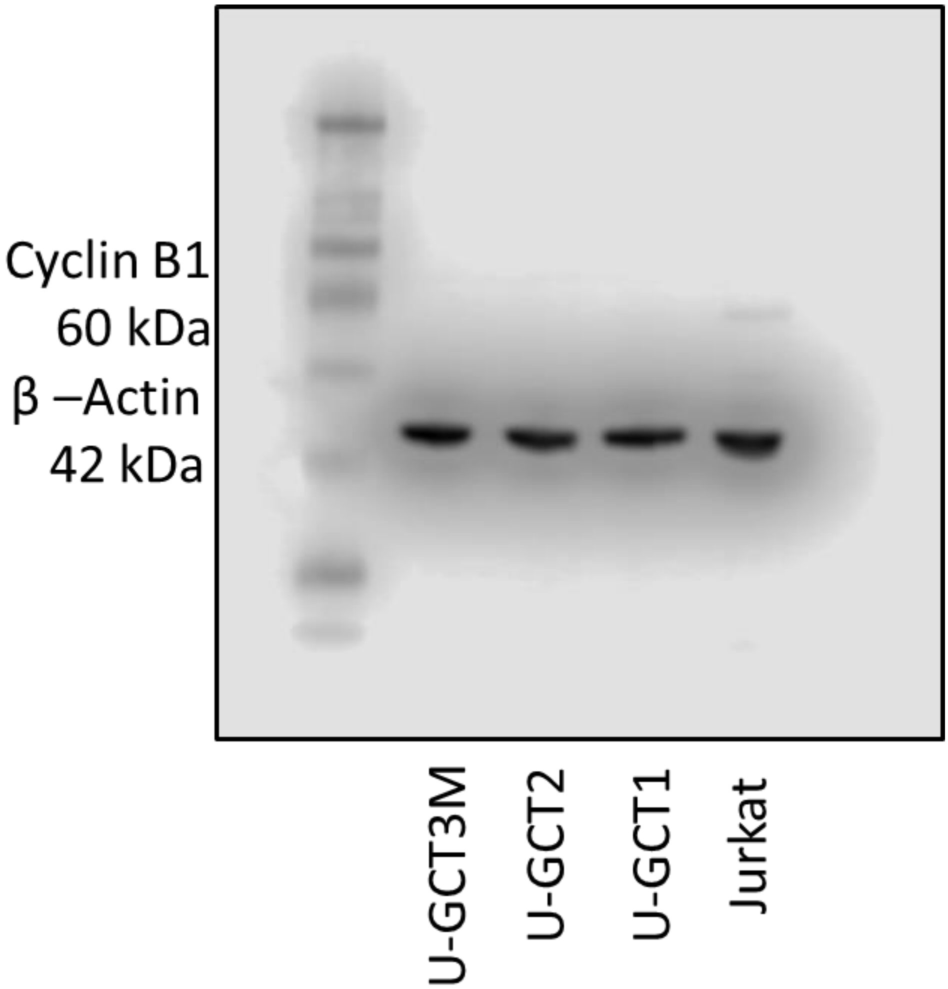

Legend Supplementary Figure 6

Whole Western-Blot detecting Cyclin B1 and β-Actin in the U-GCT cell lines and the control cell line.

Supplementary Figure 7

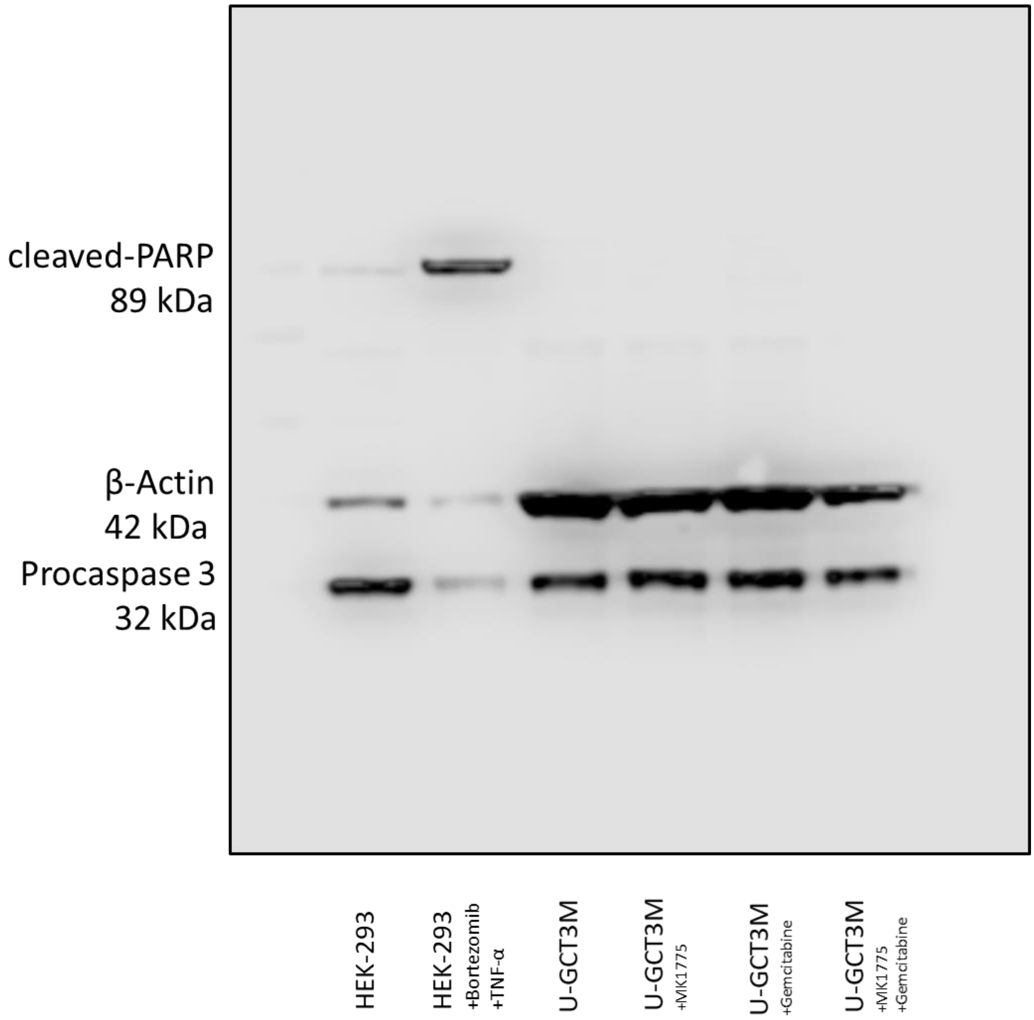

Legend Supplementary Figure 7

Whole Western-Blot detecting cleaved-PARP, β-Actin and Procaspase 3 in different treated U-GCT3M and the control cell line.

Supplementary Figure 8

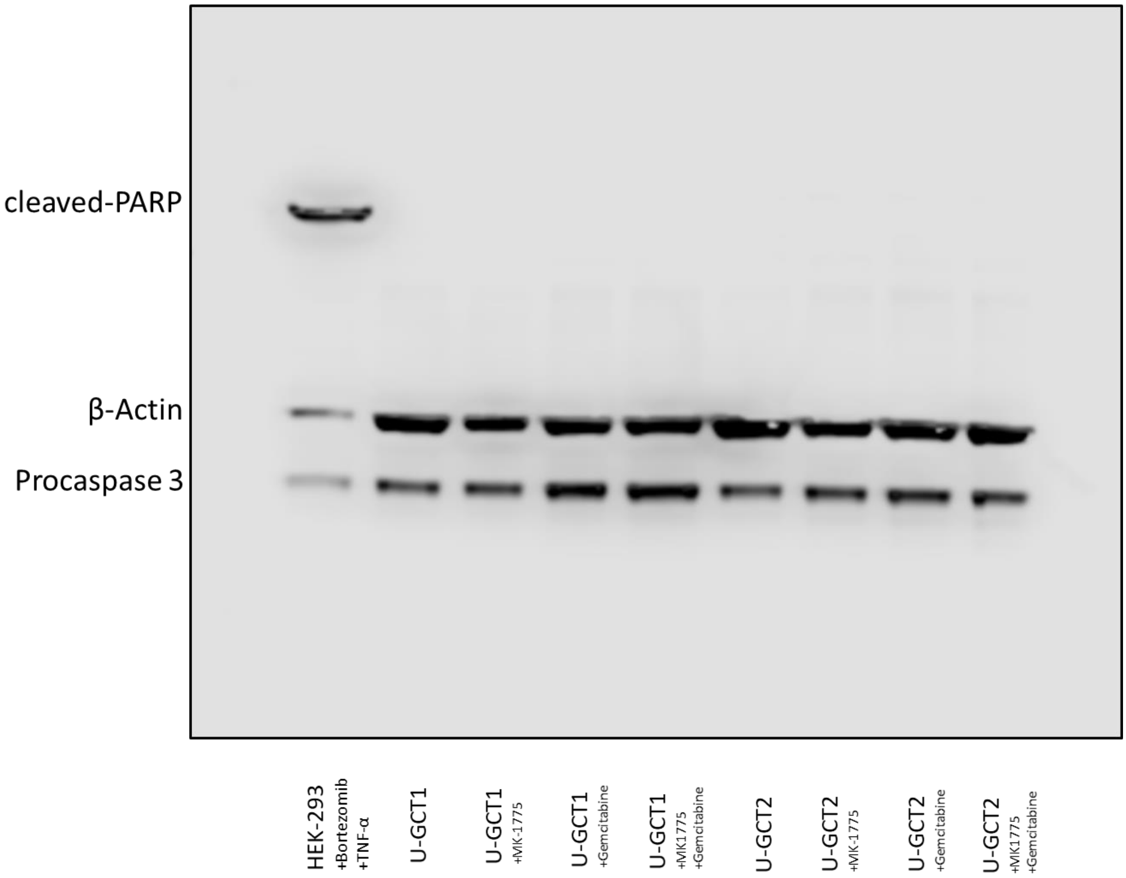

Legend Supplementary Figure 8

Whole Western-Blot detecting cleaved-PARP, β-Actin and Procaspase 3 in different treated U-GCT1, U-GCT2 and the control cell line.
